# Comparative permeability of the blood-brain barrier to albumin, DTPA, and sucrose: effects of inflammation-induced disruption

**DOI:** 10.1186/s12987-025-00748-4

**Published:** 2025-12-23

**Authors:** William A. Banks, Michelle A. Erickson, Kim M. Hansen, May J. Reed, Elizabeth M. Rhea

**Affiliations:** 1 Geriatric Research, Education, and Clinical Center, Veterans Affairs Puget Sound Health Care System, Seattle, WA 98108 USA; 2 Division of Gerontology and Geriatric Medicine, Department of Medicine, University of Washington, Seattle, WA 98104 USA

## Abstract

Blood-brain barrier (BBB) disruptions are increasingly recognized in a wide range of diseases and conditions, resulting in a need to detect and quantitate such disruptions. In humans, gadolinium (Gd)-labeled compounds including diethylenetriaminepentaacetic acid (Gd-DTPA) as detected by magnetic resonance imaging (MRI) and technetium (^99m^Tc) labeled-DTPA as detected by single photon emission computed tomography (SPECT) are commonly used, whereas ^14^C-sucrose, ^99m^Tc-DTPA, and radioactively labeled albumin are commonly used in animals. How these agents compare to one another and in different species has seldom been investigated, making comparisons between human and animal studies difficult. Here, we compared the three agents of radioactively labeled albumin, ^99m^Tc-DTPA, and ^14^C-sucrose in monolayers of human induced pluripotent stem cell (iPSC)-derived brain endothelial-like cells (iBECs), in control mice, and in mice whose BBB was disrupted by one or three doses of the inflammatory agent lipopolysaccharide (LPS). In iBECs, all three agents crossed with permeation dramatically decreasing as transendothelial electrical resistance (TEER) increased. Permeation of sucrose and DTPA were nearly identical with albumin being 10–20 times less permeable at all levels of TEER. In mice with an intact BBB, penetration of sucrose across the BBB was 2–15 times greater than DTPA, which was about 5.5 times greater than penetration of albumin (sucrose > DTPA > albumin). Uptake was about twice baseline levels for each of these agents in the 3 dose LPS (more inflamed) mice. However, in the one dose LPS (less inflamed) mice, sucrose more readily showed BBB disruption than did DTPA and DTPA more readily showed disruption than albumin (sucrose > DTPA > albumin). Linear regression analysis showed good correlations between BBB disruption as measured between albumin and DTPA (*r* = 0.783) and between DTPA and sucrose (*r* = 0.623). However, Bland-Altman analysis showed discordance between albumin and DTPA in the mice with the greatest disruption. Bland-Altman analysis also showed that disruption to sucrose was greater at any given level of inflammation than to DTPA. These subtle differences support the view that the BBB can become disrupted in different ways and that agents, which differ in size and/or structure, may vary subtly in the type or mechanism of disruption that they measure.

## Introduction

Disruptions of the vascular blood-brain barrier (BBB) are increasingly recognized as a part of many pathologies. BBB disruptions can be acute, as in stroke [1], or chronic, as in diabetes mellitus [2, 3]. BBB disruptions vary in magnitude from very large, as in multiple sclerosis [4], to minimal as in healthy aging [5]. BBB disruptions can be inborne or can be in response to secondary insults, such as oxidative stress or inflammation [6]. Disruption can also be multiphasic, as commonly occurs after traumatic brain injury [7]. Many BBB disruptions are not found throughout the brain, but are regional, affecting some regions more than others or even with some brain regions spared [2, 3].

The common occurrence of BBB disruption in so many diseases with its highly protean presentations makes it important to be able to discern disruption in humans and animal models of disease. Ideally, detection would be by a highly sensitive methodology as even the disrupted BBB retains significant barrier function compared to peripheral tissues [8]. As such, radioactive tracers are often used in animal studies and radioactive tracers or gadolinium (Gd) labeled agents are used in humans. In animal studies, sucrose labeled with either ^14^C or ^3^H or albumin labeled with ^125^I, ^131^I, or ^99m^Tc are commonly used to determine BBB integrity, with ^99m^Tc-DTPA used less frequently [9, 10]. In humans, Gd-labeled compounds including DTPA and detected by MRI or DTPA labeled with ^99m^Tc and detected by SPECT are often used [2, 11–14]. In rodents, the ability to obtain blood samples allows correction of clearance of the tracer from blood and calculation of the tracer’s pharmacokinetics measures, including brain/blood ratios, influx constants, and distribution in regions to be readily measured. Blood samples are seldom obtained in human studies and so only relative comparisons or pharmacokinetic measures based on assumptions are often made. Additionally in animal studies, the vascular space of the brain can be washed out, eliminating the vascular space as a major source of variance in the statistical analysis of BBB integrity. Because different tracers and somewhat different techniques are used in humans versus animals, it is often difficult to compare preclinical and clinical results.

Studies comparing one tracer to another (e.g., sucrose to DTPA), especially within the same animal, are not common. Such comparisons may be important as the BBB likely becomes disrupted in different ways [15]. Barrier function exists because the brain’s capillary bed has three modifications: tight junctions eliminate the intercellular channels between cells (paracellular permeation) and loss of fenestrae and reductions in pinocytosis limit transcellular permeations. The glycocalyx, largely comprised of carbohydrate polymers on the luminal surface of the BBB also contributes diffusion barrier properties, particularly limiting access of larger molecules to the endothelial plasma membrane [16, 17]. It may be that BBB disruption is loss of one or more of these barrier forming modifications and that which modification is lost relative to the others may vary with type and mechanism of disruption. Furthermore, recent evidence suggests that some Gd-labeled agents cross the BBB inside immune cells [18]. This means those agents are assessing diapedesis rather than BBB disruption.

To address these questions related to mechanism of transport, sensitivity and variability of tracers, and regional differences in permeation, we compared BBB permeability of three agents commonly used in human or animal studies of BBB disruption: ^14^C-sucrose, radioactively labeled albumin, and ^99m^Tc-DTPA. We made these comparisons in an in vitro BBB model, in control mice, and in mice exposed to low and high levels of inflammation.

### Methods

Labeling of albumin, DTPA, inulin, and sucrose: Diethylenetriaminepentaacetic acid (DTPA) labeled with ^99m^Technetium (^99m^Tc, FW 487.2 g/mol) was obtained from Radioisotope Life Sciences and sucrose labeled with ^14^C (FW 342.3 g/mol) was obtained from PerkinElmer (now Revvity). Inulin labeled with ^14^C (FW 504.4 g/mol) was obtained from PerkinElmer. When co-injected with DTPA, bovine serum albumin (BSA, FW 66,430 g/mol) was labeled with ^125^I using the chloramine-T method [19]. When co-injected with sucrose, albumin was labeled with ^99m^Tc using the stannous chloride method [20].

In Vitro BBB model: *Differentiation of iBECs*: The varied TEERs of iBECs were achieved during initial experiments that optimized seeding densities for differentiation of the GM25256 iPSC line. iBECs were differentiated from the GM25256 cell line using protocols described by Hollmann et al. [21]. These resulting iBECs have been previously characterized to have many BEC traits including expression of high TEER levels and GLUT1, CLDN5, VE-Cad, PECAM-1, ZO-1, and OCLN proteins [22]. Briefly, iPSCs were grown in E8 Medium on Matrigel in 6-well plates. When colonies were large and nearly touching, but prior to confluence, iPSCs were washed once with DPBS (Thermo Fisher Scientific) and incubated with Accutase (Stem Cell Technologies) for 3 min to yield a single cell suspension. Cells were collected via centrifugation, resuspended in E8, and counted using a hemocytometer. Cells were seeded at densities ranging from 250,000 to150,000 cells per well of a 6 plate (area = 9.5 cm^2^/well) in E8 containing 10 µM Y-27,632. Approximately 24 h after seeding, media was changed to E6 medium to induce differentiation and replaced every 24 h. Cells were differentiated in E6 medium for 4 days. Next, cells were treated with EC medium (human endothelial serum-free medium + 1% platelet-poor plasma-derived bovine serum) containing 20 ng/mL bFGF and 10 µM retinoic acid for 48 h. Following treatment, EC medium was removed, and cells were washed once with DPBS and incubated with Accutase for 15–20 min. Cells were collected via centrifugation and subsequently purified by selective adhesion onto a collagen-fibronectin matrix. Tissue culture Transwell^®^ filters (polyethylene terephthalate, 0.4 μm pore size, 0.33 cm2 surface area in 24-well format) were coated with a solution of 400 µg/mL collagen IV (Sigma-Aldrich) and 100 µg/mL fibronectin (Sigma-Aldrich) overnight. Cells were subcultured onto plates and filters at a ratio of 1 well of a 6-well plate of differentiated cells to 3–9 Transwell filters. To assess barrier properties, TEER was measured 24 h after subculture using an EVOM2 voltohmeter with an EndOhm cup chamber (World Precision Instruments). Following TEER measurement, cells were changed to EC medium without bFGF RA to induce barrier phenotype. No further media changes occurred after this point. TEER was measured approximately every 24 h thereafter. All reported TEER values are corrected for the resistance due to an empty Transwell filter. For all transwells, the maximum TEER values exceeded 300 Ω*cm^2^ on at least one day in culture, supporting that the monolayers were confluent.

Leakage was further assessed after 1–14 days post-subculture by placing in the input medium, consisting of HESFM + 1% BSA, 0.1 µCi ^14^C-sucrose and 1 million CPM ^99m^Tc-DTPA or 3 million CPM ^99m^Tc-albumin per Transwell. The 100 µL luminal (donor) chamber volume was switched to the input medium to initiate the assay, and 500 µL volumes of medium from the abluminal (receptor) chamber were collected and replaced with fresh, pre-warmed HESFM + 1% BSA medium after incubation times of 10, 20, 30, and 45 min. Of the 500 µL total volume collected, 200 µL was added to liquid scintillation vials containing 5 mL of Ecoscint™ (National Diagnostics). The vials were dark-adapted for at least 7 days. Then, the radioactivity was counted in a Tricarb beta counter (Perkin Elmer) to measure ^14^C-sucrose levels. The radioactivity in the remaining 300 µL was acid precipitated with an equal volume of 30% TCA, and the pellet (for ^99m^Tc-albumin) or supernatant (for ^99m^Tc-DTPA) were counted in a Wizard 2 gamma counter (Perkin Elmer) to measure ^99m^Tc-DTPA levels.

Treatment with LPS: Male CD-1 mice were purchased from Charles River and studied at 8–12 weeks of age. All animal studies were approved by the Institutional Animal Care and Use Committee of the Veterans Affairs Puget Sound Health Care System, an IACUC approved institution, in compliance with the Association for Assessment and Accreditation of Laboratory Animal Care International. Lipopolysaccharide (LPS) derived from Salmonella enterica subtype typhimurium (#L6511) was purchased from Sigma Chemical Company. Mice were given IP injections of approximately 0.1 ml of saline at 0, 6, or 24 h with LPS included in no injections, in only the single 0 h injection, or in all (0, 6, and 24 h) injections at a dose of 3 mg/kg LPS. Mice were studied 28 h after the 0 h injection (4 h after the last injection). Although both dosing regimens of LPS induce significant inflammation, anorexia, and weight loss in mice, the single injection induces less inflammation than the 3 injection regimen [23, 24].

Measurement of BBB permeabilities to albumin, DTPA, and sucrose: Mice 4 h after the final ip injection were anesthetized with 40% urethane and the jugular vein exposed. Mice received co-injections of either one million cpm DTPA and 10^6^ cpm albumin or 10^6^ cpm DTPA and 10^6^ dpm sucrose in 0.2 ml of lactated Ringer’s solution (LR) with 1% BSA (LR-BSA). After a specified time, the abdomen was opened and arterial blood obtained from the abdominal aorta and immediately the thorax was opened, the descending thoracic aorta was clamped, both jugular veins were severed, the left ventricle of the heart was cannulated, and 20 ml of cold LR-BSA perfused in 60 s via the left ventricle of the heart in order to wash out the vascular space of the brain. After this, brains were collected and counted in a gamma counter. The aortic blood was centrifuged at 5400 rpm for 15 min and the resulting serum counted in a gamma counter. If injected with sucrose, brains and serum were then processed with Solvable and ecoscint and radioactive levels determined in a beta counter. Brain/serum ratios are expressed in units of µl/g.

In some studies, mice were studied only at 10 min after the jugular injection of radioactivity. For multiple-time regression analysis, mice were sacrificed at 1, 2, 5, 10, 15, and 30 min after the jugular injection. In these cases, the unidirectional influx constant (Ki, in units of µl/g/min) for albumin, DTPA, or sucrose was taken as the linearity for the relation of the brain/serum ratio plotted against exposure time, calculated as

1 $$\text{Exposure time} = \left[\int_0^t\!\! {C}{p}\left(\uptau\right){d}{\uptau}\right]/{C}{p}{t}$$

where t is the time between i.v. injection and blood draw, Cp is the level of radioactivity in serum (expressed as %Inj/ml), Cpt is the level of radioactivity in serum at time t, and 𝜏 is the dummy variable for time [25, 26]. Exposure time corrects for the clearance of the compound of interest from blood. The Y-intercept measures Vi (in units of µl/g), which is the vascular space and initial luminal binding at t = 0. Ki and Vi values are reported with their standard errors and the number of mice included in the linear portion of the regression curve. Slopes and intercepts were compared statistically with program in Prism 10.0 (GraphPad Inc, San Diego, CA). The percent of the injected dose that entered each g of brain (%Inj/g) was calculated after washout of the vascular space using the equation:

2 $$\%\mathrm{Inj/g} = 100\mathrm{(cpm/g)}/\mathrm{cpm}_\mathrm{i}$$

where cpm/g is the level of radioactivity in the brain tissue, g is the weight of the brain tissue, and cpm_i_ is the amount of radioactivity injected intravenously.

Brain-to-blood efflux: Intracerebroventricular Injections: Male, 2 month old, CD-1 mice were anesthetized with 40% urethane, the scalp removed, and a hole made through the cranium and into the lateral ventricle of the brain by pushing a guarded 26 gauge needle into the skull 1 mm caudal and 1 mm lateral to the bregma [27]. One µl of LR containing approximately 10^5^ dpm of ^14^C-inulin and 3(10^5^) cpm of ^99m^Tc-DTPA was injected into this hole to a depth of 3–3.0 mm as measured from the surface of the skull. Mice were decapitated at 5, 10, and 20 min after the injection and blood collected from the arterial trunk. A zero time point was estimated by performing this intracerebroventicular injection in mice that had been dead from an overdose of urethane for 10–20 min and decapitating them 10 min later. The half-life clearance rate in min of inulin and DTPA from the brain was calculated by plotting the log of the cpm/g or dpm/g against time and multiplying the inverse of the slope of that relation by 0.301. The slope of the lines for DTPA and inulin were compared using Prism software (Graphpad Inc, San Diego, CA).

### Statistics

Means were compared using Analysis of Variance (GraphPad Inc, San Diego, CA). Regression lines with their standard errors were computed and slopes and intercepts tested for statistical differences using Prism 10.5 (GraphPad Inc, San Diego, CA). This program does not compare intercepts when slopes are significantly different. Means are reported with standard deviations.

Bland-Altman analysis is used to compare two methods of measurement. By plotting the difference between the two measures against the mean of the two methods, it can determine how well the two methods agree throughout the range of measurement as well as in distinct portions of that range. However, it would be difficult to use Bland-Altman analysis to compare albumin, DTPA, and sucrose because their means are so different. Therefore, we first normalized values by dividing them by the mean of their respective control groups, resulting in ratios. In this way, albumin, DTPA, and sucrose all had control values with a mean of unity.

## Results

### In vitro permeability

**Fig. 1:**
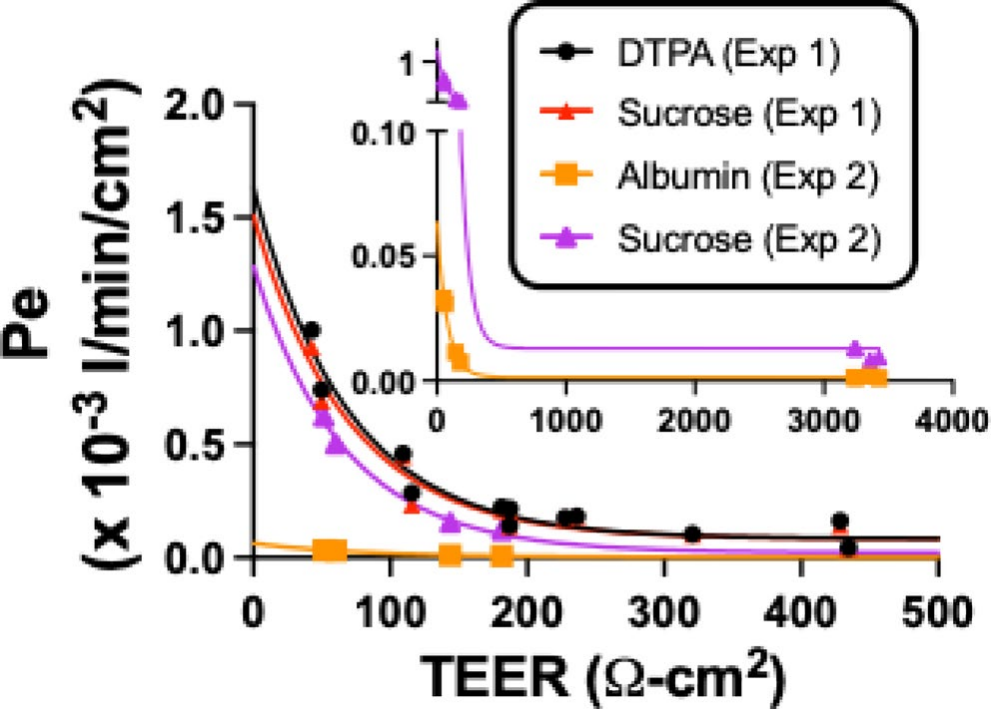
Comparison of in vitro monolayer iBEC permeabilities for ^99m^Tc-DTPA, ^14^C-sucrose, and ^99m^Tc-albumin. The permeability (Pe) for each tracer was plotted against the TEER (Ω-cm^2^) collected prior to the permeability study. Main figure shows good agreement for DTPA and sucrose when simultaneously studied (Exp 1), but with much lower permeation for albumin when simultaneously studied with sucrose (Exp 2). Inset shows full range studied for Exp 2

Figure 1 shows the relation between TEER and permeabilities for DTPA, sucrose, and albumin. For experiment 1, DTPA (*n* = 14) and sucrose (*n* = 14) were studied simultaneously by adding both to the donor chamber. For experiment 2, sucrose (*n* = 8) and albumin (*n* = 8) were studied simultaneously. The inset shows the data redrawn from experiment 2 to emphasize the albumin data. Key observations are that replication of sucrose between experiment 1 and 2 is good, showing reliability of this method, that experiment 1 shows that sucrose and DTPA have essentially identical in vitro results when studied simultaneously, and that albumin has a much lower penetration than sucrose at all TEER levels.

**Fig. 2:**
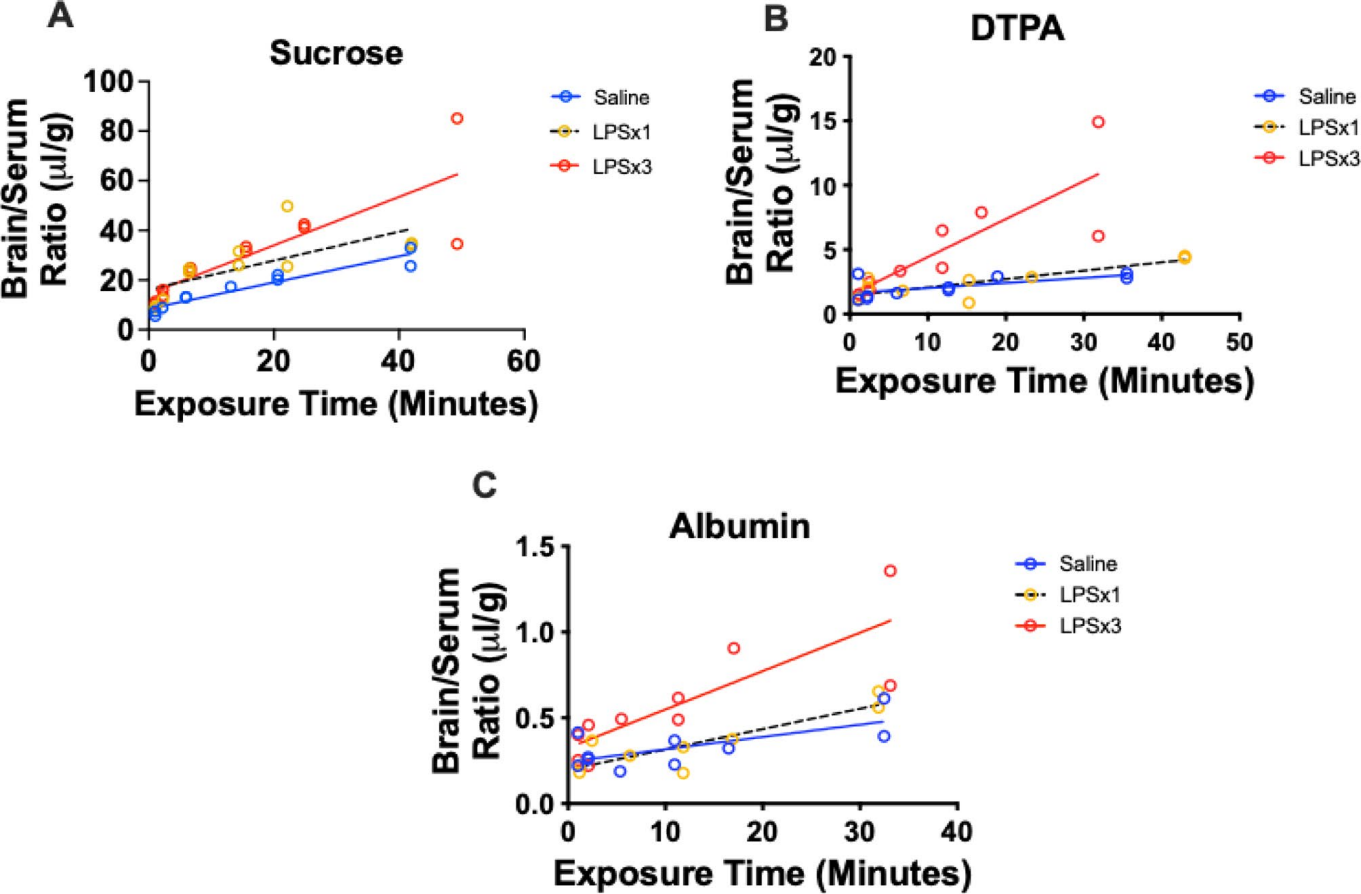
Multiple-time regression analysis for the BBB uptake of ^14^C-sucrose, ^99m^Tc-DTPA, and ^125^I-albumin. Sucrose, DTPA, and albumin all showed measurable passage across the intact BBB and modest increases with low level inflammation induced by a single injection of LPS. All showed more profound increases in BBB penetration evidencing BBB disruption with more intense inflammation as induced by the 3 injection regimen of LPS

### Time curves for LPS-treated mice

Figure 2 compares the rates of uptake by brain for sucrose, DTPA, and albumin in control and LPS-treated mice. The brain/serum ratios were higher for sucrose than for DTPA and DTPA was higher than for albumin. The Ki for sucrose (Fig. 2, Panel A) in control mice was 0.529 +/- 0.059 µl/g-min with a Vi of 8.43 +/- 1.26 µl/g (*n* = 10). The Ki for a single injection of LPS was 0.589 +/- 0.192 µl/g-min with a Vi of 16.2 µl/g (*n* = 11). The Ki for three injections of LPS the Ki was 0.975 +/-0.205 µl/g-min (about twice as high as controls) with a Vi of 14.5 +/- 4.82 µl/g (*n* = 12). For sucrose, there was no significant difference among the slopes, but intercepts differed [F [2, 29] = 5.46, *p* = 0.0097]. For DTPA (Fig. 2, Panel B), control mice had a Ki of 0.0397 +/- 0.0159 µl/g-min with a Vi of 1.62 +/-0.29 µl/g. Ki increased with a single injection of LPS to 0.0647 /- 0.0153 µl/g-min and a Vi of 1.43 +/- 0.334 µl/g and with 3 injections of LPS, the Ki was 0.294 +/- 0.0677 µl/g-min (about 7 times as high as controls) with a Vi of 1.487 +/- 1.104 µl/g (*n* = 10 for each group). For DTPA, there was a difference among slopes: F [2, 24] = 12.38, *p* = 0.0002. For albumin, (Fig. 2, Panel C) the Ki for controls was 0.00718 +/- 0.00264 µl/g-min with a Vi of 0.245 +/- 0.0431 µl/g (*n* = 10), for a single LPS injection the Ki was 0.0119 +/- 0.00255 µl/g-min with a Vi of 0.197 +/- 0.0438 µl/g (*n* = 9), and for 3 injections of LPS the Ki was 0.0224 +/- 0.00535 µl/g-min (about 3 times higher than the Ki for controls) and a Vi of 0.325 +/- 0.0893 µl/g (*n* = 10). For albumin, there was a difference among these slopes: F [2, 23] = 4.25, *p* = 0.027. Baseline or control Ki for sucrose was 15 times higher than that for DTPA and 82 times higher than that for albumin; the control for DTPA was about 5.5 times higher than the control Ki for albumin. All three showed increased leakiness of the BBB with the 3 injection dose of LPS.

### Single time point analysis

**Fig. 3:**
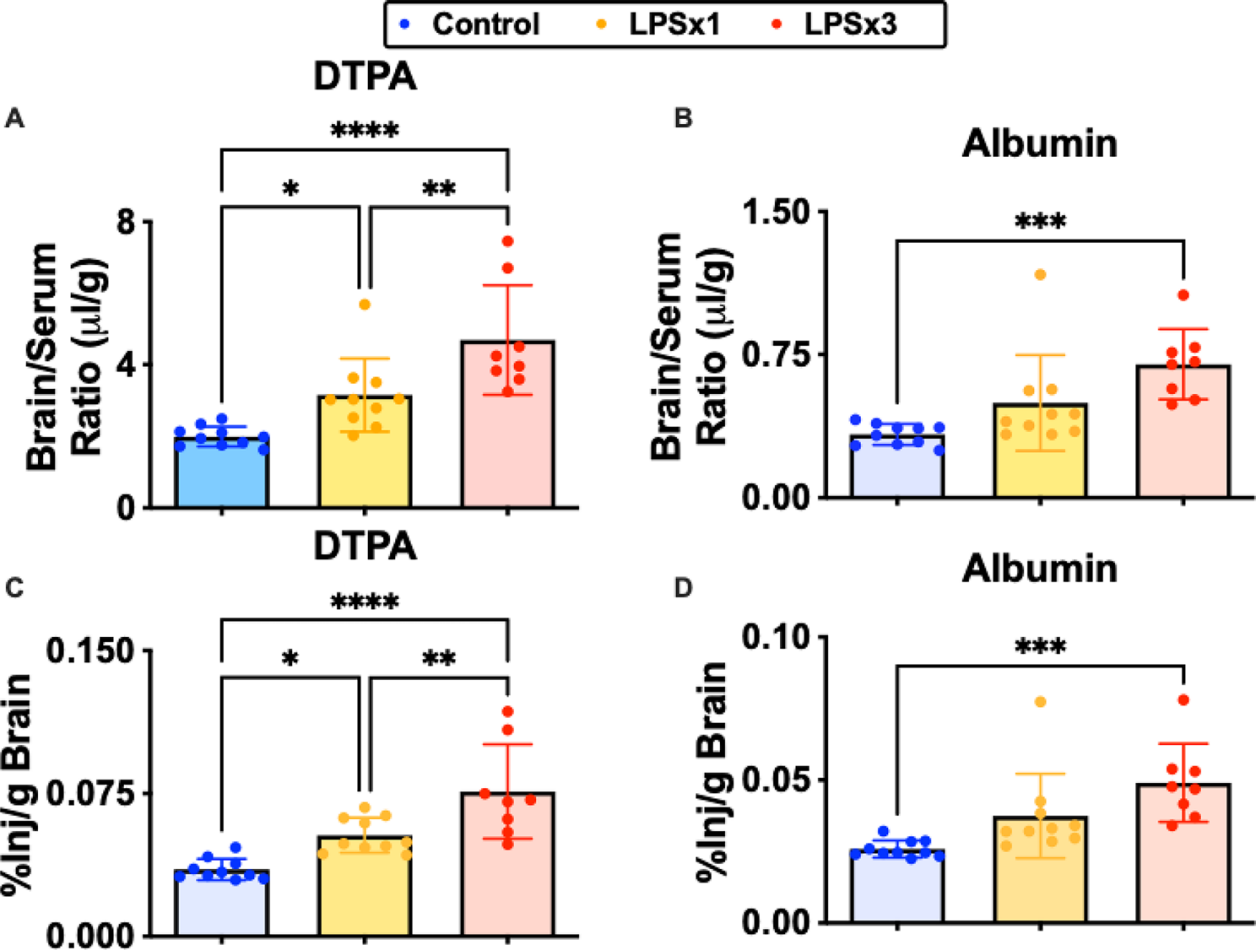
Comparison of DTPA and albumin uptake across the BBB in control and inflamed mice. Results are for mice studied at a single time point (10 min). Panel A shows brain/serum ratio results for DTPA and panel B for Albumin. Panels C and D show results as expressed as percent of the injected dose per g of brain. Control represents mice not given LPS, LPSx1 are mice that received a single injection of LPS, and LPSx3 are mice that received the 3 injection regimen of LPS. *P < 0.05, **p < 0.01, ***p < 0.01, ****p < 0.001

Figure 3 shows the brain/serum ratios for mice which received simultaneous jugular injections of DTPA (Panel A) and albumin (Panel B) with a 10 min circulation time. DTPA control values were about 5 fold higher than those for albumin. DTPA was more sensitive than the simultaneously injected albumin in detecting BBB disruption as DTPA showed disruption at the single dose of LPS as well as at the three doses. However, the three dose regimen of LPS more than doubled leakiness values for both albumin and DTPA, increasing them to almost the same degree: 111% (albumin) vs. 136% (DTPA). Figures 3C and D show the percent of the injected dose that entered brain (%Inj/g-brain), which is a measure often of interest in studies of drug delivery. The %Inj/g-brain takes into account peripheral pharmacokinetics (volume of distribution and half-life in blood) as well as rate of BBB permeation. The %Inj/g-brain for albumin (0.026) was nearly the same as for DTPA (0.035) for control mice, illustrating the effect of a smaller volume of distribution and longer half-life for albumin in comparison to DTPA. With three injections of LPS, albumin increased 81% to 0.0490%Inj/g-brain and DTPA increased 117% to 0.0760%Inj/g.

**Fig. 4:**
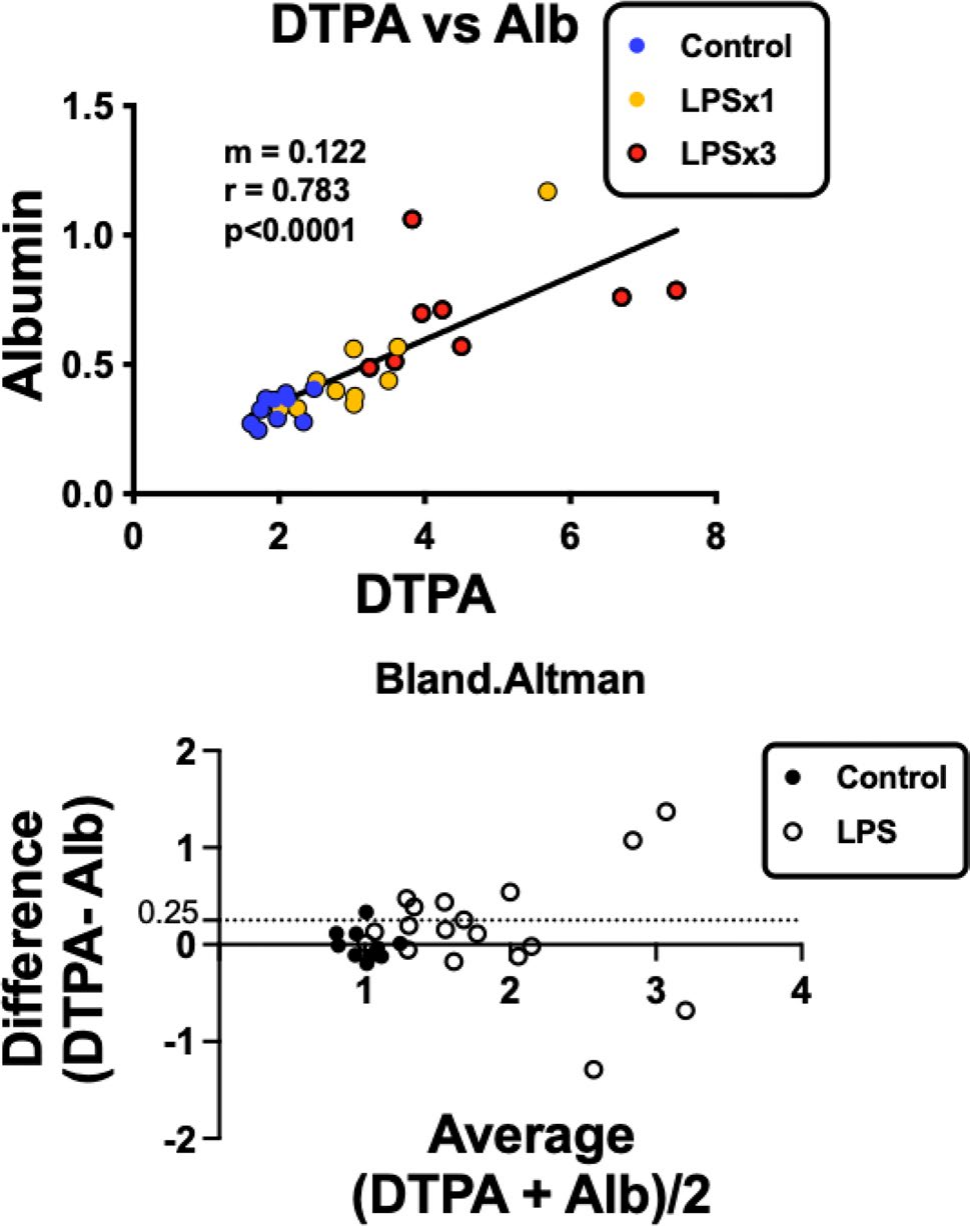
Comparison of DTPA and albumin uptake across the BBB studied 10 min after iv administration. Upper panel shows regression analysis for control mice and those given one (LPSx1) or three (LPSx3) injections of LPS. Lower panel shows Bland-Altman analysis

Fig. 4 upper panel shows the correlation between DTPA vs. albumin brain/serum ratio (µl/g) for the single time point data. This shows a high degree of correlation between the two substances with a correlation coefficient of 0.783. Bland-Altman analysis is superior to linear regression for determining the relation between two measures across their range (Fig. 4, lower panel). As expected because of our normalization step, control values were zero for the differences between DTPA and albumin and 1.0 for the average of DTPA and albumin. For X axis values up to about 2 (that is, up to a doubling of BBB permeability), the Difference (y axis) showed a value of about 0.25 (dashed line), consistent with DTPA being more permeable in this range of disruption than albumin. For values greater than 2, the main feature is a high degree of variance, suggesting that DTPA and albumin measures no longer agree on the magnitude of disruption. In some cases, values are below the line, showing albumin as more permeable than DTPA and others are positive, showing DTPA to be more permeable than albumin.

**Fig. 5:**
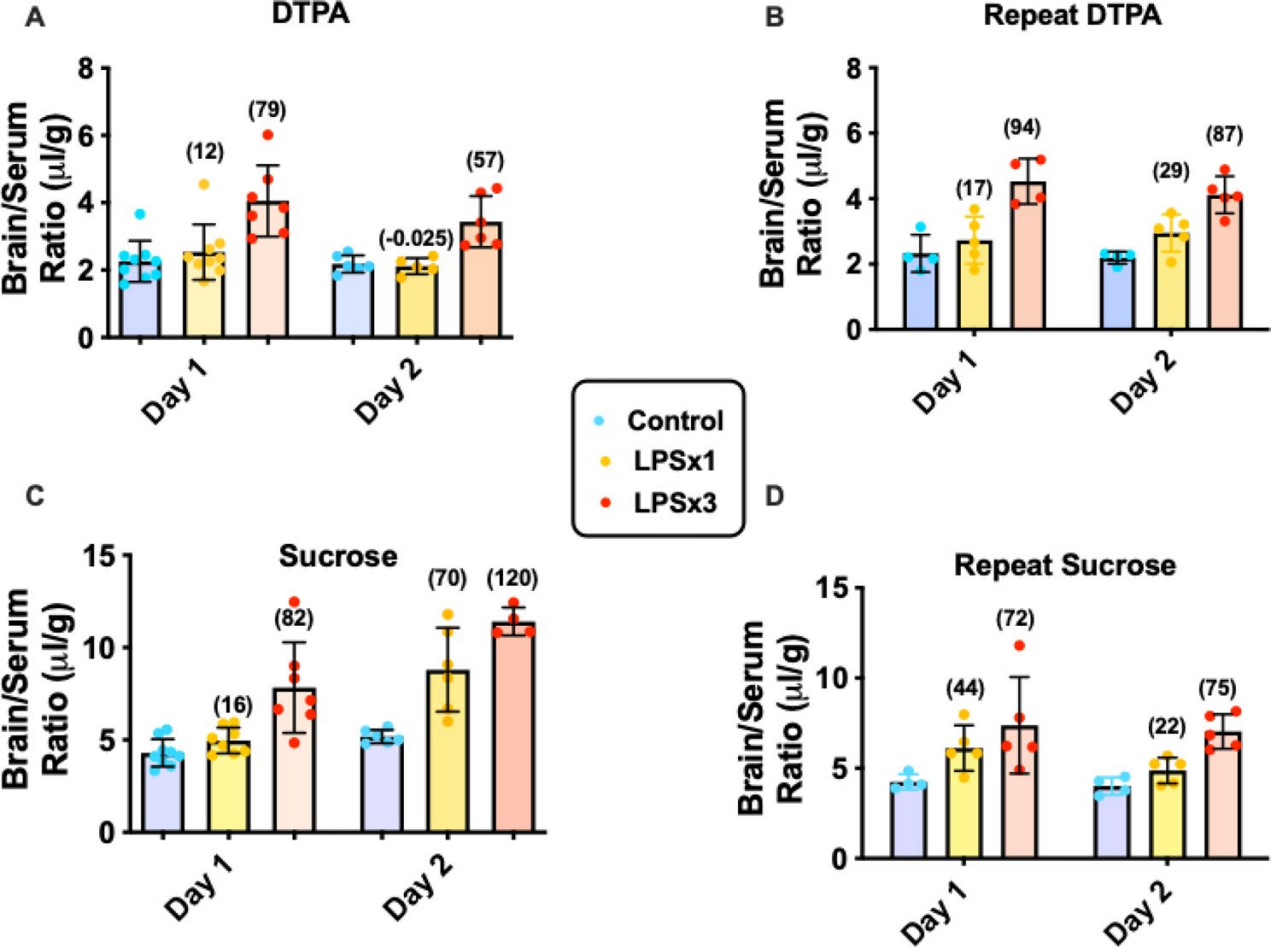
Comparison of simultaneously administered DTPA and sucrose with experiment repeated over 4 sessions (Day 1, Day 2, Repeat Day 1, Repeat Day 2). Control mice are shown in blue, mice receiving a single injection of LPS are shown in yellow (LPSx1), and mice receiving 3 injections of LPS are shown in red (LPSx3). Results show degree of agreement and variance among repeats and between DTPA and sucrose. % change relative to respective Control (blue) is in parentheses

**Fig. 6:**
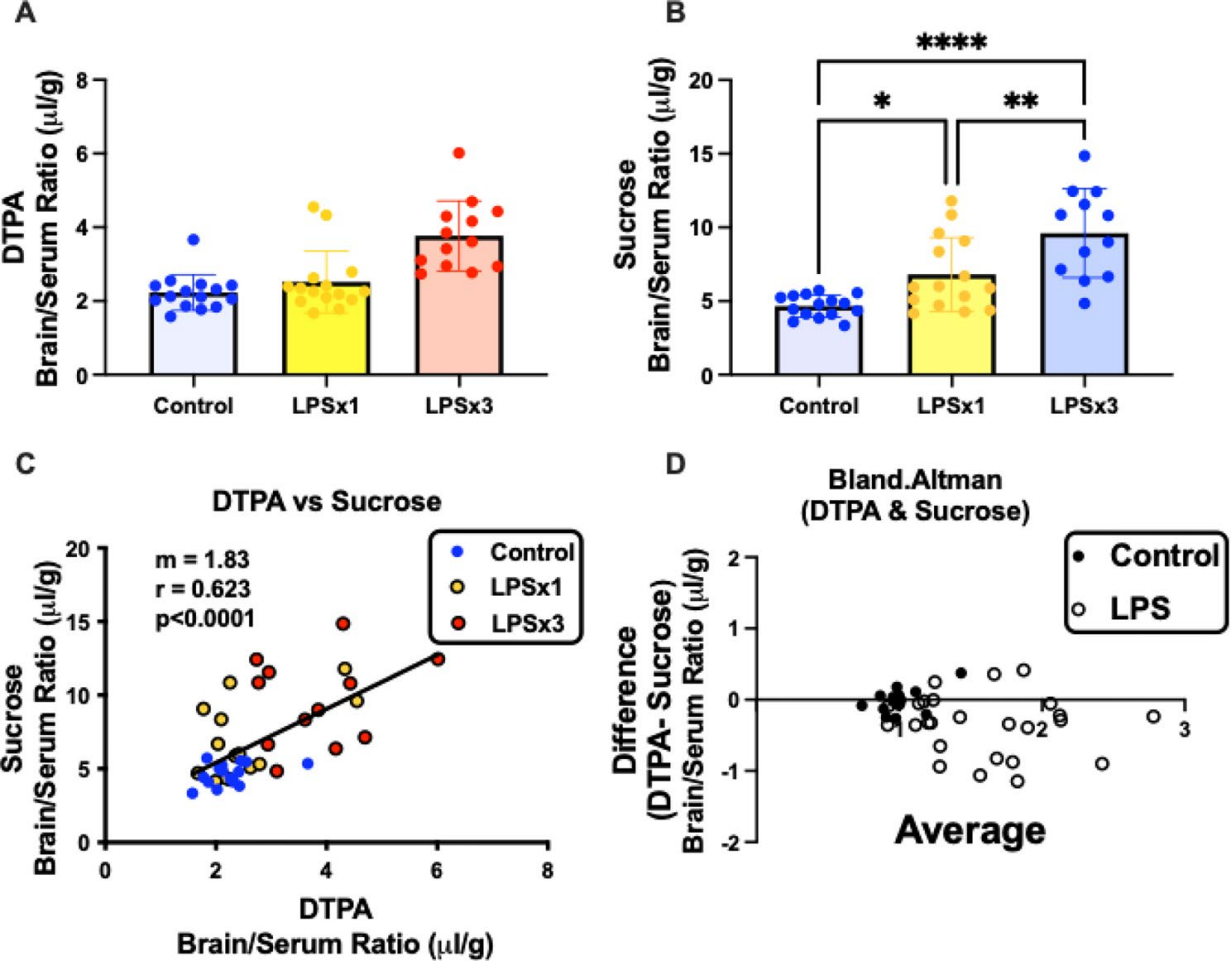
Combined data from Fig. 5 comparing controls, single injection of LPS (LPSx1), and three injections of LPS (LPSx3) for DTPA and sucrose. Panel **A** shows BBB disruption to DTPA after 3, but not after 1, injection of DTPA. Panel **B** shows disruption to sucrose after either 1 or 3 injections of LPS. Panel **B** shows regression analysis for DTPA vs. sucrose results. Panel **D** Bland-Altman analysis shows leakage is progressively greater for sucrose than for DTPA as leakage increases

**Fig. 7:**
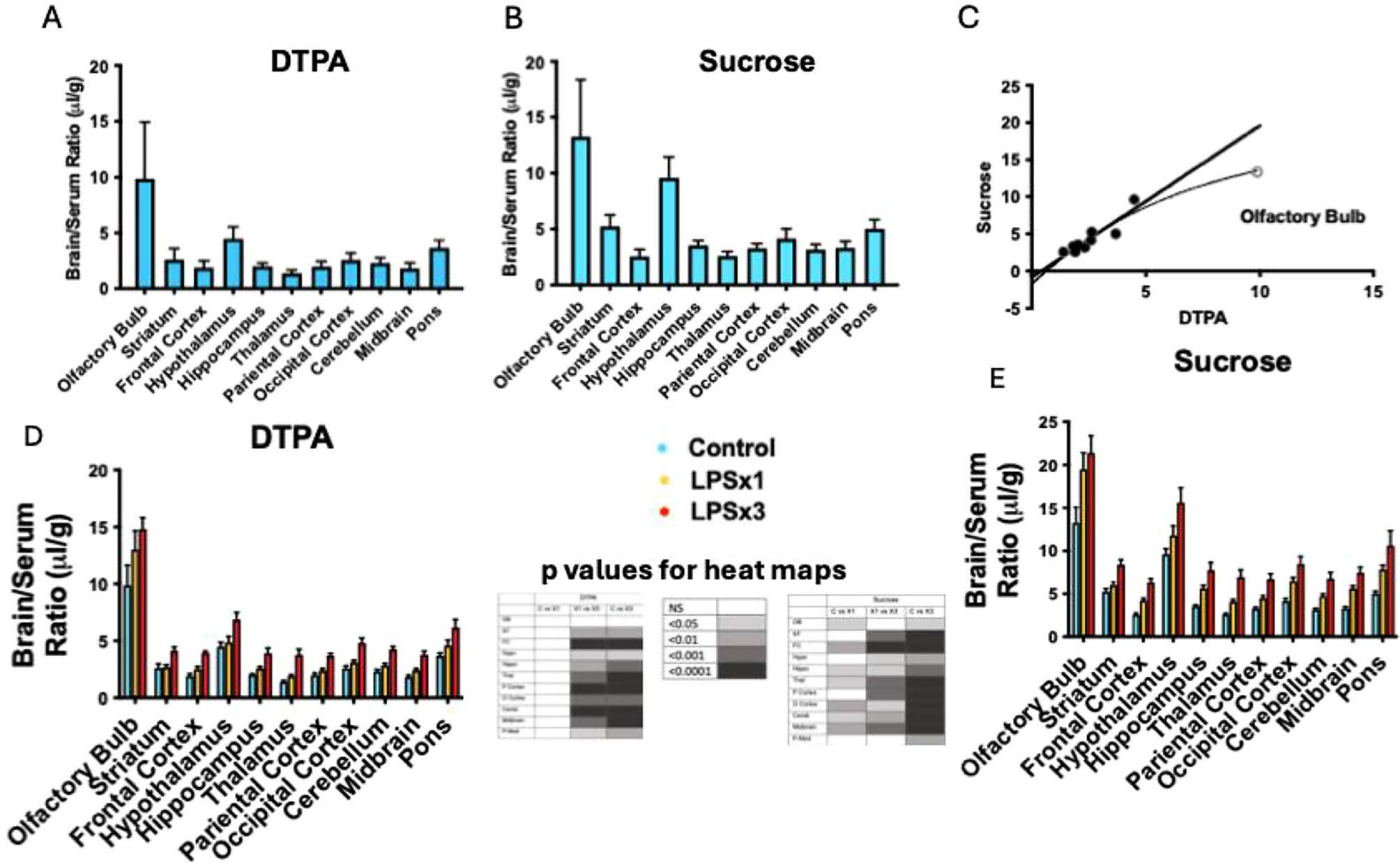
Brain region permeability to DTPA and sucrose. Panel **A** shows penetration of DTPA into various brain regions in control mice. Panel **B** shows penetration of sucrose into various brain regions in control mice. Panel **C** shows good agreement between DTPA and sucrose in control mice across regions, but with a proportionately lower value for olfactory bulb. Panel **D** shows a single injection of LPS (LPSx1) did not disrupt the BBB to DTPA, but that three injections of LPS (LPSx3) increased uptake by all brain regions except the olfactory bulb. Panel E shows that a single dose of LPS disrupted the BBB in many brain regions, while three injections of LPS disrupted all regions of the BBB. Legend and p value heat map shown for panels **D** and **E**

Figs. 5, 6, 7 compare BBB permeability for sucrose and DTPA under control (baseline) and inflammatory conditions. Variance of permeability of both baseline and in response to LPS was tested for DTPA and sucrose by doing the study over two days (Figs. 5A and C) and then repeating this paradigm a few months later (Figs. 5B and D). Thus, 4 sets of experiments (Day 1 and Day 2 of the first study and Day 1 and Day 2 of the repeat study a few months later) can be compared for permeability and its variance. In some respects, the data in Fig. 5 shows a high level of consistency. For example, baseline brain/serum ratios for DTPA have a narrow range between 2.18 and 2.33 µl/g and for sucrose between 4.02 and 5.18. The 3 dose injection of LPS reliably increased permeability between 57% and 94% (X = 79.2 +/- 16; CV = 20.2%) for DTPA and between 72% and 120% (82 +/-22.2; CV = 27%) for sucrose. In other respects, the data showed variability. Single injection LPS effects ranged from a small decrease (-0.025%) in once case for DTPA to a robust increase (70%) in one case for sucrose. The mean percent increase for the four measures of single dose LPS was 14.5 +/- 12 (CV = 85%) for DTPA and 38 +/-24.5 (CV = 64%) for sucrose The increase in sucrose was greater than that for DTPA in 5 of the 8 cases and lower in 3 of the cases and there was not a correlation between the percent increase for DTPA vs. the percent increase for sucrose (data not shown).

In order to improve statistical power for a comparison of DTPA vs. sucrose, the data from the 4 experimental sessions were combined (Fig. 6). Figures 6A and B show that control values for sucrose were again higher than DTPA values. In this experiment, one injection of LPS did not disrupt the BBB to DTPA but did to sucrose. Three injections of LPS increased DTPA permeation by 69% and sucrose by 107%. The correlation coefficient between DTPA and sucrose (Fig. 6C) was less robust than for DTPA vs. albumin at 0.623, indicating less agreement between these two measures of BBB disruption. Bland-Altman analysis (Fig. 6D) shows that LPS treated mice tended to have negative scores for Difference, indicating greater increases in sucrose values in comparison to DTPA values as BBB disruption increased.

Figure 7 compares permeability to simultaneously administered sucrose and DTPA for brain regions 10 min after iv injection. Panels A and B show permeability of the BBB to DTPA and sucrose, respectively, in mice not treated with LPS (control mice). For each brain region, sucrose values were greater than those for DTPA, although the olfactory bulb appears to be proportionately less elevated than other brain regions as it departs from the linear relation for the other brain regions (Fig. 7C). Separate one-way ANOVAs were performed for each brain region for DTPA and for sucrose, comparing control, single LPS injection, and three LPS injections. No brain region had a statistically significant increase in permeability to DTPA after a single injection of LPS, but all regions except the olfactory bulb had a significant increase after 3 injections of LPS. For sucrose, seven regions had an increase in permeability after one injection of LPS and all regions had an increase after 3 injections.

Brain-to-blood Efflux: Sequestration and transport can affect the efflux rate of a substance from brain, which in turn can affect the substances retention. We compared the efflux of DTPA to that of the gold standard, inulin [28]. The half-time clearance for DTPA from brain was 37.4 min and for inulin was 28.9 min (data not shown). There was no statistical difference between these two slopes. This shows that DTPA appears to be neither sequestered by brain tissues nor is it likely transported out of the brain by a saturable system.

## Discussion

Here, we compared the abilities of albumin, DTPA, and sucrose to detect BBB disruption in an in vitro model of the human BBB and following no, low or high inflammatory insults in a mouse model in vivo. The iBEC data showed over a wide range of TEER values that DTPA and sucrose had nearly identical permeabilities. In comparison, albumin had much lower permeation at all TEER values. In vivo, we examined BBB disruption induced by a milder inflammatory insult with a single injection of LPS and by a more robust inflammatory insult with three injections of LPS. Treatments with LPS activate the innate immune system, producing very profound losses of body weight and alterations in blood and brain cytokine levels, demonstrating that it is a potent stimulator of inflammation and neuroinflammation [23, 24]. We washed out the vascular space of the brain so that the remaining radioactivity was either sequestered by the BBB or had crossed into the brain parenchyma.

We found that all 3 compounds crossed the in vitro BBB. DTPA and sucrose transfer rates were indistinguishable from each other, whereas at all TEER values albumin was less permeable than sucrose. In vivo, all three compounds again had demonstrable penetration of the BBB with penetration of albumin being much lower than the other two compounds. Unlike in vitro, however, the in vivo BBB distinguished between DTPA and sucrose, with sucrose uptake exceeding that of DTPA by about two-fold. These hierarchies held for both the control and the disrupted BBB. The difference between DTPA and sucrose is not expected because they were so similar in vitro and because they have very similar molecular weights. However, these results support previous findings of Miah et al. [29] who showed that sucrose labeled with ^14^C overestimated sucrose permeation across the BBB by about two-fold. The exact cause of this overestimation is unclear but is at least partially explained by impurities in the sucrose mixture and a higher lipid solubility of ^14^C-sucrose. These results also highlight that in vitro and in vivo models can give different results. For example, we have previously found that in the in vitro monolayer model, exposure to LPS alters tight junction protein localization [24], whereas ultrastructural examination after in vivo exposure to LPS shows no tight junction alterations, but a host of other changes including increased vesicularization [30].

In general, linear regression showed good agreement between the permeability of DTPA and those of albumin and sucrose. At the higher inflamed state as induced by the 3 injection regimen of LPS, all compounds clearly showed statistically significant increases in permeability, demonstrating BBB disruption. At the less inflamed state as induced by the single injection of LPS, DTPA more readily detected BBB disruption in comparison to albumin and sucrose was more sensitive than DTPA. Bland-Altman analysis is superior to simple linear regression in comparing two measures as it can show the variance or degree of agreement between the measures as well as whether the relation between the measures changes over the range of the measures. Here, Bland-Altman confirmed that DTPA values were slightly higher than those for albumin at modest (less than two fold) increases in BBB leakage. At higher levels of BBB leakage, the relation between DTPA and albumin deteriorated. This suggests that different mechanisms of BBB disruption could dominate DTPA vs. albumin leakage at higher levels of BBB disruption. Bland-Altman analysis showed that sucrose tended to leak into brain more readily than DTPA and did so increasingly as BBB disruption progressed. Overall, these results show that albumin, DTPA, and sucrose are all sensitive markers for BBB disruption, but that there are subtle differences and disagreements in the measures of those disruptions.

Some of these subtle differences are seen in the inflammation-induced permeabilities to DTPA vs. sucrose in Fig. 5. Because DTPA and sucrose were co-injected, results from the same animal can be directly compared (e.g., DTPA Day 1 to Sucrose Day 1 (in Panels A and C) or Repeat DTPA Day 1 to Repeat Sucrose Day 1 (in Panels B and D)). The comparisons between DTPA and sucrose are aided by the variable inflammatory responses to LPS among replicas. In some cases, the agreement between the increases in DTPA and sucrose are very good (e.g., 12% vs. 16%; 79% vs. 82%; 29% vs. 22%) and in other cases very divergent (e.g., -0.025% vs. 70%; 57% vs. 120%). Percent increases for sucrose were greater than for DTPA in 5 cases and less than for DTPA in three cases. These variances seem too great to be caused by technical issues such as variability in radioactive detections and suggests that DTPA and sucrose may not cross the BBB by totally identical mechanisms.

The percent of the injected dose taken up per g of brain (%Inj/g) for albumin and DTPA is compared in Figs. 3C and D. This measure of %Inj/g is often of more interest for drug development than the brain/serum ratio as it shows how much of the drug has entered the brain. The %Inj/g takes into account not only the rate of entry into brain, but also the peripheral pharmacokinetics. Thus, the difference between DTPA and albumin is diminished because of albumin’s long half-life in blood and its small volume of distribution. For both DTPA and albumin, the increase in brain uptake was about two fold. Although DTPA has a molecular weight similar to many drugs that have effects on brain, BBB-penetrating drugs tend to have a high lipid solubility and to enter the BBB rapidly by transmembrane diffusion. Therefore, disruption of the BBB is likely to have lesser effects on their brain uptake because the magnitude of uptake by transmembrane diffusion is so much larger than that caused by disruption.

We might have obtained different results had we studied a different cause of disruption. The magnitudes of disruption are known to vary greatly among diseases and conditions, with low levels in aging [5] and Alzheimer’s disease [13] and greater levels in diabetes mellitus [2] and multiple sclerosis [4]. Additionally, the cell biology varies as disruptions induced by LPS are mediated by prostaglandins [24], whereas those for traumatic brain injury induced by blast are related to nitric oxide [31]. Finally, the mechanism by which increased permeability to a probe can occur is varied and includes loss of tight junctions, increased pinocytosis, adsorptive transcytosis, reinduction of fenestrae, increased immune cell penetration, and loss of the glycocalyx [15–18, 32, 33]. With all of these variables amongst disease states, it can be hoped that probes will emerge with selectivity for the various types or aspects of disruption, thus aiding in the diagnosis of BBB dysfunctions.

In conclusion, we compared the uptake of three commonly used radioactive markers for BBB disruption: albumin, DTPA, and sucrose. All showed a very low, but measurable, uptake in both the in vitro and in vivo models. In vitro, sucrose and DTPA had nearly identical values of permeation, whereas albumin was much lower. In vivo, all three agents had a very low, but measurable, permeation of the BBB in control mice. Albumin, as expected for a large molecule, showed a much lower uptake than DTPA or sucrose, but in contrast to the in vitro model, sucrose uptake was higher than the similar sized DTPA. The BBB was very resilient to disruption by inflammation, showing only about a two-fold increase in the disrupted state vs. the control state when measured as single time point data in the most inflamed group of mice. In less inflamed mice, DTPA was more sensitive than albumin in detecting disruption and sucrose more sensitive than DTPA. As assessed by simple linear regression, there was good agreement among the compounds as to magnitude of disruption. However, discordances between the measures of BBB disruption for DTPA compared with albumin and DTPA compared with sucrose suggest that there may be some subtle differences in the mechanisms by which these compounds cross the BBB. Whereas each of these radioactively labeled compounds detected subtle disruptions of the BBB, sucrose tended to be the most sensitive agent. Discordances between sucrose and DTPA and between DTPA and albumin suggest that different mechanisms of BBB disruption are being differentially detected under certain conditions such as varying levels of inflammation.

## Data Availability

No datasets were generated or analysed during the current study.
